# Atherogenic index of plasma and its cardiovascular risk factor correlates among patients with type 2 diabetes in Uganda

**DOI:** 10.4314/ahs.v23i1.54

**Published:** 2023-03

**Authors:** William Lumu, Silver Bahendeka, Ronald Wesonga, Davis Kibirige, Ronald Mutebi Kasoma, Emmanuel Ssendikwanawa

**Affiliations:** 1 Department of Medicine Mengo Hospital Kampala Uganda & Texila American University George Town Guyana; 2 Department of Medicine, Mother Kevin Post-Graduate Medical School, Uganda Martyr's University; 3 School of Statistics & Planning Makerere University Uganda; 4 Department of Medicine, Uganda Martyr's Hospital Lubaga, Kampala; 5 Clinical Epidemiology Unit, Makerere University College of Health Sciences, Uganda; 6 Clinical Epidemiology and Biostatistics, Clinical Epidemiology Unit, Makerere University College of Health Sciences, Uganda

**Keywords:** Atherogenic index of plasma, Type 2 diabetes, cardiovascular risk factors, Uganda

## Abstract

**Background:**

Atherogenic Index of Plasma (AIP) is a reliable predictor of coronary artery disease. There is paucity of data on AIP and its correlates among patients with type 2 diabetes (T2D) in Uganda.

**Objective:**

To assess the profile of AIP and its cardiovascular risk factor correlates among patients with T2D in Uganda.

**Methods:**

This was a cross-sectional study conducted in 8 health facilities with established T2D clinics in Central-Uganda. The study enrolled 500 patients aged 40 to 79 years. Data was collected on socio-demographic characteristics, lipid profile and glycated haemoglobin (HbA1c). The AIP was derived using log (triglycerides/high-density cholesterol) and further categorised as low cardiovascular disease (CVD) risk if the AIP was <0.1, intermediate risk (0.1-0.24) and high risk (≥0.24). Cardiovascular risk factors were defined according to international guidelines.

Stata version 14 was used to analyse data, Pearson correlation analyses were conducted. Statistical significance was set at p<0.05.

**Results:**

There were 389(77.4%) females with a mean age of 55.07±8. 979 years. Low-risk was found in 43.6%, intermediate risk in 20.2% and high risk in 36.2% of the participants. AIP significantly correlated with waist circumference (r=0.1095, p<0.0147), waist-hip ratio (r=0.1926, p<0.001), Casteri Risk Index I (r=0.506, r=<0.001), Casteri Risk Index II (r=0.246, p<0.001) and atherogenic coefficient (r=0.186, p<0.001). Insignificant correlation was observed between AIP and fasting blood sugar (r=0.017, p=0.7042), HBA1C (r=0.0108, p=0.8099) and diabetes duration (r=0.0445, p=0.32)

**Conclusions:**

AIP is significantly elevated and correlated with cardiovascular risk factors in patients with T2D. In clinical management, this may be a useful tool in risk stratifying patients with T2D.

## Introduction

Diabetes mellitus (DM) is an established risk factor for cardiovascular disease (CVD)^1^, and patients with DM are at a two to four-fold higher cardiovascular risk, including myocardial infarction, unstable angina, stroke, and heart failure^2^. All of the above have arisen interest in CVD preventive strategies by the use of non-invasive methods, such as risk scores.^3^ The most common approach is to consider DM as a CVD equivalent and, therefore, to treat patients with DM in a similar way to those who required secondary CVD prevention^4^. However, this approach has been disputed as all patients with T2D do not have the same CVD risk^5^,^6^ and since other potentially important factors within the context of DM, such as DM duration, presence of albuminuria, and comorbidities, should be taken into consideration. Moreover, diabetic dyslipidaemia which is major orchestrator of CVD is characterised by elevated triglyceride(TGs), reduced high density cholesterol(HDL) and small LDL particles 2 Diabetic dyslipidaemia in T2D is comprised of quantitative, qualitative and kinetic abnormalities of the lipid parameters 7 In clinical practice, it is the quantitative abnormality that is routinely assessed through estimation of lipid parameters which may be normal or subnormal missing the opportunity to tackle qualitative and kinetic abnormalities 8 hence predisposing patients to CVD. The early detection of lipid abnormalities with their management can mitigate the risk of atherogenic cardiovascular and cerebral vascular disorders in T2D patients^9^. Timely detection of lipid abnormalities can be achieved through quantitative determination of lipid variables and atherogenic cardiovascular indices^8^. Qualitative akinetic abnormalities characterised by small LDL particles would be indirectly measured by Lipoprotein(a) assays^10^, but these are not routinely done due to cost and unavailability of test. LDL particle size can be assessed with Atherogenic Index of Plasma. Atherogenic Index of Plasma (AIP)defined as the base 10 logarithm of the ratio of plasma triglyceride (TG) to high density lipoprotein (HDL)^11^, has been used to assess for small LDL particles and plasma atherogenicity^11^ .AIP is a major predictor of atherosclerosis and coronary heart disease and reflects the relationship between protective and atherogenic lipoproteins^12^, AIP is thus an indicator of LDL particle size that is not determined by the usual lipid profile.

It can be easily derived from the usual lipid profile which is done widely among T2D patients in Uganda at no added cost. AIP can be used to stratify T2D patients so that those at high CVD risk can be started on drugs with proven cardiovascular benefit^13^ such as Sodium Glucose Co Transporter 2 inhibitors (SGLT2) or Glucagon like peptide 1 agonists (GLP-IA) as stipulated by several International guidelines such as the American Diabetes Association Standards of Care on cardiovascular disease and risk management 2022^13^. Currently, the majority of medicine and diagnostic tests essential in the management of type 2 diabetes and CVD are unavailable and unaffordable in Uganda^14^. So, prevention is key though screening with cheap and available tests such as the lipid profile and its derivative, the Atherogenic Index of Plasma.

Whereas the statin dose intensity and use of ant platelet agents is clear for those who have had a CVD event^13^ it is not clear for those who are asymptomatic, hence there is need for guidance in this regard so that meagre resources can be used sparingly in a poor resource setting. This study looked at AIP and its cardiovascular risk factor correlates in a Ugandan T2D population.

Knowledge of the profile of AIP and its cardiovascular risk factor correlates is key for CVD risk stratification^11^,^15^, so that T2D patients can be started appropriately on drugs with proven cardiovascular benefit to prevent or manage CVD 13 at primary level in our poor resource setting.

Additionally, markers of obesity such as body mass index, waist circumference, waist hip ratio and atherogenic indices have been shown to correlate with a high CVD risk^9^. Therefore, proof of this correlation with AIP among T2D patients in Uganda will be useful in predicting CAD risk in centres where a lipid profile can't be performed. Therefore, the current study was conducted to assess the profile of Atherogenic Index of Plasma and its cardiovascular risk factor correlates among patients with T2D in Central Uganda.

## Materials and Methods

### Study Design and Population

This was a cross-sectional study conducted in eight (8) diabetes clinics in Central-Uganda namely; Entebbe grade B hospital, Mengo Hospital, Naguru Hospital, Kasangati Health Center IV, Wakiso Health Center IV, Mpigi Health Center IV, Mityana Hospital and Kawolo Hospital. Detailed description of the methodology has been published elsewhere^16^. The study was approved by Mengo Hospital Research and Ethics Committee (Approval number MH/REC/100/9-2019) and was registered by Uganda National Council of Science and Technology (Registration number HS2738). We obtained administrative clearance letters from heads of participating hospitals. Written informed consent was obtained from each study participant. We followed the Declaration of Helsinki during the execution of this study.

Data was collected from 500 patients with T2D aged between 40 and 79 years who were consecutively selected from the diabetes registers. Eligible patients were given unique identification numbers derived from the health facility code and registration number.

Socio-demographic data on age, sex, level of education, employment status and smoking were collected by trained study nurses through face-to-face interviews.

Anthropometric, clinical and laboratory measurements were conducted. Details of these measurements have been published elsewhere.^16^

### Atherogenic Index of Plasma

Atherogenic Index of Plasma (AIP) was calculated from Log10(TG/HDLc) ratio. Low CAD risk was when AIP was -0.3-<0.1, medium risk 0.1 -<0.24 and high CAD risk ≥0.24.^17^

### Cardiovascular Risk factors

Cardiovascular risk factors in T2D were defined according to W.H.O 18 and American Diabetes Association Standard of Care Guidelines^13^. American Diabetes Association Standard of Care 2021 targets for cardiovascular risk factors among T2D were used to define CVD risk factors among our study population^13^. Elevated glycated haemoglobin was defined ≥ 7%, systolic blood pressure≥ 140mmHg, diastolic blood pressure≥90mmHg, low-density lipoprotein cholesterol ≥ 2.56mmol/l(100mg/dl)^13^. Additionally, elevated fasting blood sugar was ≥7mmol/, total cholesterol≥5.12mmol/l and triglyerides≥ 1.7mmol/L^13^. A high waist circumference was defined as ≥88cm for women and ≥102cm for men. We took a waist-hip ratio ≥0.88(women) and ≥0.95(men)and a body mass index >25Kgm2 to be elevated 18. A duration of ≥ 10years, gender was taken as a CVD risk factors^3^.

Smoking was defined as daily cigarette smoking, family history of premature coronary heart disease was defined as history of CHD in first degree relatives aged < 55 years for males and <65 years for females^3^.

Castelli Risk Index 1(CRI-1) was estimated as: TC/HDLc ratio, A low risk was defined as <3.5^15^. Castelli Risk Index II(CRI-II) was determined as LDL-c/HDLc ratio. A low-risk was defined <3.0^15^. Atherogenic Coefficient (A.C) was calculated as (TC-HDLc)/HDLc or (Non-HDLc)/HDLc ratio A low risk was defined as < 3.0^15^.

### Statistical analysis

The data collected was checked for completeness, coded and entered into EpiData manager version 4.6 and exported to STATA version 14. Continuous variables were described using the mean and standard deviation. Categorical variables were expressed as frequencies and percentages and were compared using Chi-square tests. The correlation between AIP and cardiovascular risk factors was determined by Pearson chi-square correlation analyses. A p-value of less than 0.05 was considered statistically significant.

## Results

### Socio demographic characteristics

Out of the 500 study participants, 389 (77.4%) were females and the mean age of the participants was 55.07years±8.979. One hundred eight seven (37.4%) were aged between 50 and 59 years. Three hundred twenty-three (64.6%) of the study participants were urban residents. About 442(88.4%) did not have history of premature coronary heart disease death in the family. The majority 448(89.6%) did not take alcohol and 491(98.2%) did not smoke. Thirty-five (7%) of the participants were on lipid lowering medications by the time of our study. About three hundred and sixty-seven (73.4%) of the participants had had DM for less than 10 years while 364(72.8%) were taking ant hypertensive medications. Details are shown in Table 1.

**Table 1:** Socio-demographic and clinical characteristics of participants

Variable	Female (n=389)	Male(n=111)	All(N=500)	Mean±SD
	n (%)	n (%)	n (%)	
**Age Group**				
40–49	114(29.31)	30(27.03)	144(28.8)	
50–59	141(36.25)	46(41.44)	187(37.4)	
60–69	105(26.99)	28(25.23)	133(26.6)	
>70	29(7.46)	7(6.31)	36(7.2)	
**Mean age**				55.076±8.978
**Residence**				
Urban	252(64.78)	71(63.96)	323(64.6)	
Rural	137(35.22)	40(36.04)	177(35.4)	
**History of Premature** **CHD death**				
Yes	31(7.97)	5(4.50)	36(7.20)	
No	337(86.63)	105(94.59)	442(88.40)	
Don't know	21(5.40)	1(0.90)	22(4.40)	
**Alcohol intake**				
Yes	21(5.4)	13(11.11)	34(6.8)	
No	358(92.03	90(81.08)	448(89.6)	
Quit	10(2.54)	8(7.2)	18(3.6)	
**Smoking**				
Yes	1(0.26)	0.00	1(0.2)	
No	386(99.23)	105(94.59)	491(98.2)	
Quit	2(0.51)	6(5.41)	8(1.6)	
**Cholesterol Lowering** **Medicine use**				
Yes	24(6.17)	11(9.91)	35(7)	
No	365(93.83)	100(90.09)	465(93)	
**Duration of DM**				
<10years	288(78.47)	79(21.53)	367(73.4)	
>10years	101(75.94)	32(24.06)	133(26.4)	
**Use of Hypertensives**				
Yes	291(74.81)	73(65.771)	364(72.8)	
No	98(25.19)	38(34.23)	136(27.2)	

### Profile of the Atherogenic Index of Plasma

**Figure 1:**
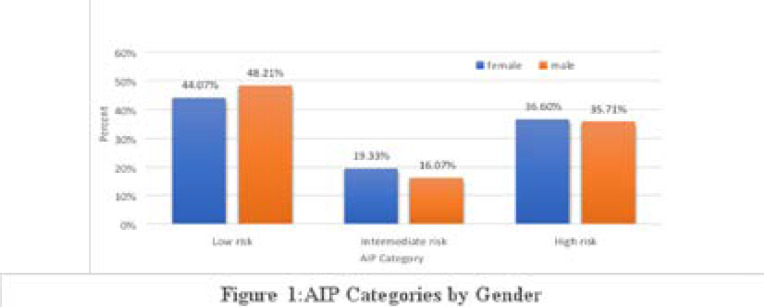
AIP Categories by Gender

Out of 500 participants,218(43.6%) had low, 101(20.2%) moderate and 181(36.2%) high AIP. The mean AIP was 0.177±0.413. There was no significant gender difference across all categories of AIP as shown in figure 1.

### Cardiovascular risk factors among study participants

Three hundred and seventy-nine (75.8%) of the study participants had a BMI ≥25Kg/m2 with a mean of 29.78 Kg/m2 ±5. 778. Two hundred and eighty-four (56.8%) had elevated waist-hip ratio though there was no significant difference in mean waist circumference between females and males though men had a higher mean waist-hip ratio 0.942±0. 106. Elevated systolic blood pressure and diastolic blood pressure was found among 211(42.2%) and 158(31.6%) of participants respectively. However, the mean combined blood pressure across all the study participants, was 137.53/85.62mmhg.

Elevated total cholesterol was found among 290(58%) of the study participants with a mean value of 5.405mmol±1.319 while 393 (78.6%) had elevated LDL with a mean of 3.502mmol/l±1. 181.Seventy-two percent (72%) of the participants had elevated glycated Hemoglobin (HbA1C≥7%). About two thirds had fasting hyperglycemia (fasting blood sugar ≥ 7mmol/l). The mean DM duration was 6.952 ±6.383 years.

Regarding cardiovascular indices,357(71.4%) of the participants had an elevated CRI I and there was no significant gender difference(p=0.818). Two hundred eighty-one (56.2%) of the participants had their A.C raised. Similarly, there was no gender difference noted in this parameter(p=0.524). More details about the CVD risk factors are shown in tables 2 and 3.

**Table 2:** Anthropometric and laboratory measurements of Participants (N=500)

Variable	Female (n=389)	Male(n=111)	All(N=500)	p-value
	n (%)	n (%)	n (%)	
**Body mass Index**				
<18Kg/m2	1(0.26)	0.00(0)	1(0.2)	0.56
18–24.99Kg/m2	70(17.99)	34(30.63)	104(20.8)	
25–29.99Kg/m2	135(34.7)	44(39.64	179(35.8)	
≥30Kg/m2	183(48.59)	11(9.91)	200(40)	
**Waist Circumference**				
<88.8cm	200(51.47)	100(90.09)	300(60)	
≥88.8cm	189(48.59)	11(9.91)	200(40)	
**Waist Hip Ratio**				
<0.88	156(40.17)	60(54.05)	216(43.2)	
≥0.88	233(59.90)	51(45.95)	284(56.8)	
**SBP**				
<140mmHg	237(60.93)	52(46.85)	289(57.8)	0.008
≥140mmHg	152(39.07)	59(53.15)	211(42.2)	
**DBP**				
<90mmhg	273(70.18)	69(62.16)	342(68.4)	0.109
≥90mmhg	116(29.82)	42(37.84)	158(31.6)	
**T.C**				
<5.13mmol/l	147(37.79)	63(56.76)	210(42)	<0.001
≥5.13mmol/l	242(62.21)	48(43.24)	290(58)	
**HDL-c**				
≥1.3 mmol/l	342(87.92)	87(78.38)	233(46.6)	<0.001
<1.3mmo/l	47(12.08)	24(21.62)	267(53.7)	
**TG**				
<1.685mmol/l	159(40.87)	62(55.86)	221(44.2)	0.005
≥1.685mmo/l	230(59.13)	49(44.14)	279(55.8%)	
**LDL-c**				
<2.56mmol/l	66(16.97)	41(36.94)	107(21.4)	<0.001
≥2.56mmol/l	323(83.03)	70(63.06)	393(78.6)	
**CRI I**				
<3.5	110(28.35)	32(29.46)	143(28.6)	
≥3.5	278(71.65)	79(70.54)	357(71.4)	0.818
**CRI II**				
<3.3	282(72.94)	86(76.79)	369(73.8)	
≥3.3	105(27.06)	26(23.21)	131(26.2)	0.415
**AC**				
<3.0	167(43.04)	52(46.43)	219(43.8)	
≥3.0	221(56.96)	60(53.57)	281(56.2)	0.524
**FBS**				
<7mmol/l	125(32.13)	37(33.33)	162(32.4)	0.812
≥7mmo	264(67.87)	74(66.67)	338(67.6)	
**HbA1C**				
<7%	112(28.79)	28(25.23)	140(28)	
≥7%	277(71.21)	83(74.77)	360(72)	0.46
**AIP**				
Low risk (<0.11)	171(44.07)	54(48.21)	225(45)	
Intermediate risk (0.11-<0.24	75(19.33)	18(16.07)	93(18.6)	
High risk≥0.24	142(36.6)	40(35.71)	182(36.4)	

**n** frequency, **SBP**: Syst olic blood pressure, **DBP**: Diastolic blood pressure, **HbA1c**: Glycated haemoglobin, **T.C**: Total cholesterol, **HDL-C**: high density lipoprotein cholesterol, **TG**: Triglycerides, **LDL-C**: Low density lipoprotein cholesterol, **CRI I**: Casteri Risk Index I, **CRI II**: Casteri Risk Index II, **AC**: Atherogenic Coefficient: **FBS**: Fasting Blood Sugar: **HBA1C**: Glycated Hemoglobin, **AIP**: Atherogenic Index of Plasma

**Table 3:** Mean anthropometric, clinical and laboratory measurements of study participants

Variable	Mean (± SD)		
	Female	Male	All
**BMI** (kg/m^2^)	30.25 (5.76)	28.15 (5.58)	29.78 (5.78)
**W.C** (cm)	88.40 (12.66)	88.88 (11.83)	88.50 (12.44)
**W. H. R**	0.89 (0.09)	0.94 (0.11)	0.90 (0.09)
**SBP** (mmHg)	136.19 (19.51)	141.65 (19.71)	137.54 (19.66)
**DBP** (mmHg)	85.19 (11.42)	87.12 (10.72)	85.62 (11.29)
**HBA1C** (%)	8.9 (2.85)	8.97 (2.73)	8.92 (2.82)
**FBS** (mmol/l)	9.59 (5.14)	9.26 (3.81)	9.51 (4.88)
**T.C** (mmol/l)	5.54 (1.29)	4.92 (1.32)	5.41 (1.32)
**HDL-C** (mmol/l)	1.36 (0.37)	1.23 (0.35)	1.33 (0.37)
**LDL-C** (mmol/l)	3.63 (1.16)	3.07 (1.17)	3.50 (1.18)
**TGs** (mmol/l)	2.06 (1.06)	2.14 (1.76)	2.08 (1.25)
**CRI-1**	4.29 (1.38)	4.25 (1.54)	4.28 (1.42)
**CRI-11**	2.79 (1.03)	2.57 (1.02)	2.74 (1.03)
**AIP**	0.18 (0.44)	0.18 (0.321)	0.18 (0.413)
**A.C**	3.18 (1.28)	3.25 (1.16)	3.28 (1.43)
**Diabetes Duration** (years)	6.92 (6.21)	7.07(6.99)	6.95 (6.38)

**BMI**: Body Mass Index: **W.C**: Waist Circumference: Waist Hip Ratio, **SBP**: Systolic Blood Pressure, **DBP**: Diastolic Blood Pressure, **HBA-IC**: Glycated Hemoglobin, **FBS**: Fasting Blood Sugar, **TC**:Total Cholesterol, **HDL**: High Density Lipoprotein Cholesterol, **LDL-C**:Low Density Lipoprotein Cholesterol, **TGs**: Triglycerides, **CRI 1**: Casteri Risk Index I, **CRI II**: Casteri Risk Index II, **AIP**: Atherogenic Index of Plasma: **AC**: Atherogenic Coefficient:

### Correlation between Atherogenic Index of Plasma and cardiovascular risk factors

**Figure 2:**
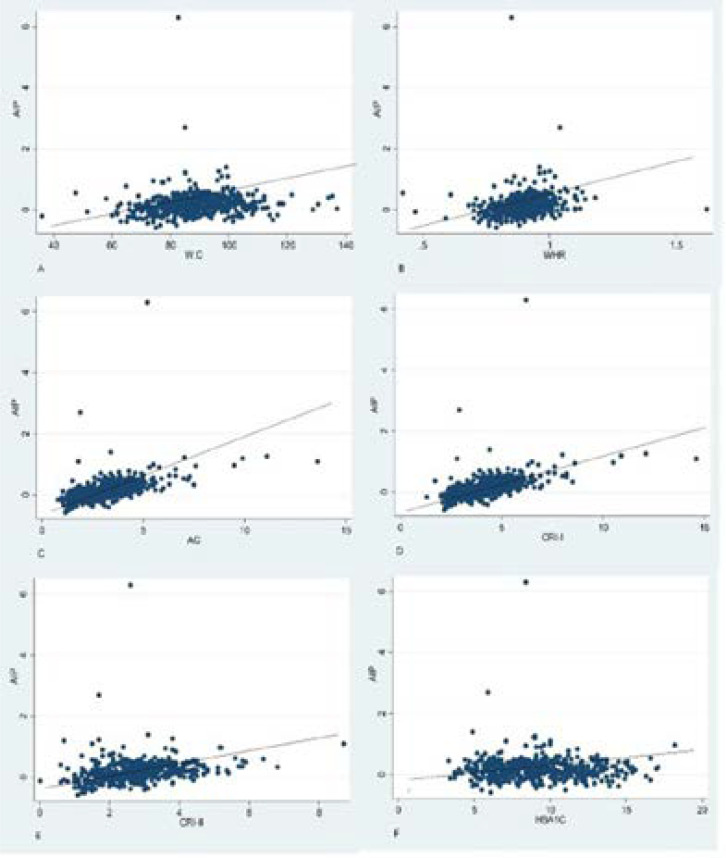
Correlation of AIP with A) Waist Circumference, B) Waist Hip Ratio, C) Atherogenic Coefficient D) Casteri Risk Index I E) Casteri Risk Index II F) Glycated Hemoglobin WC=Waist Circumference, WHR=Waist-Hip Ratio, CRI I=Casteri Risk Index I, CRI II=Casteri Risk Index II, HBA1C=Glycated Hemoglobin

We performed Pearson's correlation analysis to investigate the correlation between AIP and cardiovascular risk factors. AIP positively and significantly correlated with waist circumference (r=0.1095, p<0.0147), waist-hip ratio (r=0.1926, p=0.0000), CRI I (r=0.506, r=<0.001), CRI II (r=0.246, p<0.001) and A.C(r=0.186, p<0.001). Furthermore, weak and insignificant correlation was noted between AIP and sex (r=0.003, p=0.995), BMI (r=0.0349, p=0.436), total cholesterol (r=0.0691, p=0.012), fasting blood sugar (r=0.017, p=0.7042), glycated haemoglobin (r=0.0108, p=0.8099), LDL (r=0.0810, p=0.0705, DM duration (r=0.0445, p=0.32). Details are depicted in table 4 and figure 2.

**Table 4:** Correlation between AIP and Cardiovascular Risk Factors

Parameter	r	P -value
Age	0.0746	0.0957
Male Sex	0.0003	0.9950
BMI	0.0349	0.436
Waist circumference	0.1095	0.0147
Waist Hip ratio	0.1976	<0.001
Fasting blood sugar	0.017	0.7042
Glycated Hemoglobin	0.018	0.8095
Systolic blood pressure	0.0457	0.3055
Diastolic blood pressure	0.0093	0.8361
Duration of diabetes	0.0445	0.3209
Total cholesterol	0.0691	0.123
LDLc	0.0810	0.07055
Casteri Risk Index I	0.185	<0.001
Casteri Risk Index I	0.127	<0.004
Atherogenic Coefficient	0.186	<0.001

**BMI**=Body Mass Index, **LDLc**=Low Density Lipoprotein cholesterol, **r**= Pearson's correlation coefficient

## Discussion

In this cross-sectional study, we determined the profile of atherogenic index of plasma and its cardiovascular risk factor correlates among patients with T2D in 8 rural and urban clinics in Central Uganda. Our study showed 56.4% of the participants had intermediate and high AIP (atherogenic risk) with no significant difference in distribution across gender. The current study showed that a significant proportion of our patients with T2D had poorly controlled cardiovascular disease risk factors.

Our study showed that AIP was positively and significantly correlated with waist circumference, waist- hip ratio, CRI-I, CRI-II and A.C. Weak and insignificant correlation was noted between AIP and sex, BMI, total cholesterol, fasting blood sugar, glycated haemoglobin and duration of diabetes.

Similar results were shown in one study among adult Africans living with HIV infection by Steve Raul Noumegni et al where 52.4% had intermediate and high atherogenic risk 19.

In a large population-based survey conducted over 7000 subjects in rural Uganda,42% of the individuals had high and intermediate atherogenic risk (20). The differences in study design, population sample, nature of patients studied and specimen collected could explain the differences in the findings between the two studies.

In a study conducted by S. Bhardwaj et al, AIP, CRI 1, CRI II and AC were significantly elevated in angiographically confirmed cases of CAD and contributed significantly to the CAD risk with AIP contributing 31% followed by CRI 1 with 20%, AC 17% and CRI II^11^. It is not surprising that these cardiovascular indices are elevated in our T2D patients since diabetes portends a very high coronary heart disease risk. Our study has shown a significant correlation between AIP and CRI I, CRI II and AC. These indices signify a high plaque density in the coronary arteries^11^. Therefore, their elevation among our study participants indicates a high CAD risk that needs urgent tackling in our setting.

Our findings are consistent with a study done in Nigeria by Bolarinwa et al that found a high AIP in 31.4% of diabetic and hypertensive patients attending a tertiary hospital 17. This study is similar to ours in that they were both cross-sectional hospital-based studies with similar patients studied. However, there was a gender difference in terms of the atherogenic risk with females more at risk than men that was attributed to the higher number of women recruited in the study. Our study had a higher number of women but we didn't show any gender difference in the risk probably due to the fact that with a mean age of 55.07±8.979years, women in our study were post-menopausal. It has been shown that before menopause, estrogen is cardiovascular protective and hence in the premenopausal period men tend to have a higher CVD risk than women but this advantage cancels out after menopause 3. In a similar study by Olamoyegun et al, AIP showed no significant difference between sexes in predicting cardiovascular disease risk 15..

Consistent with earlier studies 12,17,21–23, AIP positively and significantly correlated with waist circumference and waist-hip ratio. We didn't not show significant relationship between AIP and BMI similar to a study by Steve Raoul Noumegni et al^19^. Waist-hip ratio is a better marker of visceral adiposity than BMI which does not differentiate between body fat and lean mass^24^. Visceral adiposity is associated with increase in free fatty acid levels that cause insulin resistance by reducing insulin signalling at the target tissues. Insulin resistance is a driver of cardiovascular disease 23.

In our study population, more than 50% had diabetic dyslipidaemia characterised by a high LDL (mean 3.502mmol/l±1.181), high triglyceride (mean 2.078mmol/l±1.24) and low HDL (mean 1.33mmol/l±0.372), this type of dyslipidaemia typical of diabetes is highly atherogenic and is not surprising that 56.4% of our study participants had a high plasma atherogenicity as determined by the AIP. This is in tandem with several other studies 12,17,23,25.

Contrary to other studies^12^,^17^,^23^, we didn't show a significant relationship between AIP and LDL despite 78.4% of the study participants having elevated LDL probably the two measures assess different abnormalities in an individual. Whereas the importance of LDL cholesterol in the development of atherosclerosis is well documented, and it being the principal target for prevention of CHD, the heterogeneity of the LDL particles has been recently elucidated 24 Different forms of LDL that include small dense LDL particles and oxidized LDL have been described and these constitute the kinetic abnormalities of LDL other than the quantitative abnormality that is routinely assessed by the usual lipid profile. In one study in Canada, small dense LDL particles predicted the rate of ischaemic heart disease independent of LDL cholesterol, TGs, HDL cholesterol, apo B and the TC/HDL ratio. The same study showed that LDL diameter predicts ischaemic heart disease risk more accurately than traditional lipid variables. Therefore, AIP assesses the small dense LDL particles while the absolute LDL measurement determines the quantitative abnormality. Our finding underpins the utility of assessing for both among our patients with the AIP very crucial in those patients who seem to have normal or low LDL values^24^.

We have earlier shown that 65.8% of patients with T2D have a high 10 year predicted ASCVD risk^16^ as determined with the Pooled Cohorts Risks Equation and the current study has shown that 56.4% of our patients have elevated AIP. The 10 -year predicted ASCVD risk assesses the global ASCVD risk i.e., coronary heart disease, ischaemic stroke and peripheral arterial disease risk while AIP is mainly a predictor of coronary artery disease^26^, which is the main cause of morbidity and mortality among the CVD spectrum in patients with T2D. These findings can be explained by high LDL levels among our patients (mean LDL was 3.5±1.181) and only 7 % of the patients were taking lipid lowering drugs by the time of our study. Therefore, the significant elevation of AIP in more than half of the patients shown in our study means that there is an urgent need for use of high dose statin, SGLT2 inhibitors, GLPA1 agonists, ant platelet agents and other life style changes for primary prevention of CVD^13^

In our study, AIP was not significantly correlated with systolic and diastolic blood pressure which was in tandem with a study done by Manoj Kumar et al assessing the association of AIP with haemodynamic variables in normotensive and never treated hypertensive subjects. In that study, AIP didn't correlate with arterial Blood pressure but correlated directly and indirectly with arterial stiffness^27^. In one study, AIP correlated with systolic blood pressure and diastolic blood but there was no significant difference(p=0.06) between hypertensive and non-hypertensive groups which was due to a higher number of normotensive participants^22^. It should be noted that the majority of our patients had well controlled hypertension which could have explained the insignificant correlation. The current study did not show significant correlation between AIP and measures of glycaemia namely fasting blood sugar and glycated haemoglobin. The reasons for this remain unclear to us. However, our findings are similar to those found by James B Meiggs et al in a study in which there was no association between glycemic control and prevalent cardiovascular disease among 1,539 patients with Non-Insulin Dependent Diabetes Mellitus^28^

Contrary to our study, Zhang H et al in a large prospective population-based cohort study among 10060 Chinese, showed that an HbA1C of 7-8% was significantly associated with a high CVD risk in patients with baseline ASCVD risk score≥10%^29^. The difference in outcomes could be due to a larger sample size, follow up period and difference in CVD risk assessment method.

While hyperglycemia is evidently associated with microvascular complications, its role in the causation of large vessel atherosclerosis is controversial^28^.

Despite the fact that our patients' mean glycated hemoglobin was 8.919±2.818% which is strongly related to CVD^30^, we didn't show this relationship as causation of ASCVD by hyperglycemia is time dependent^31^, i.e., more evident when duration of diabetes is ≥10years. In the current study, 73.4% of our patients had had diabetes for less than 10 years with a mean duration of 6.952 ±6.38 years. Additionally, this being a cross sectional study, causal relationship could not be established.

The major strength of this study is that it assessed atherogenic AIP and its cardiovascular risk factor correlates among patients with T2D for the first time in Uganda. Secondly, the study was conducted in one region of the country with limited ethnic variation. However, there are some limitations. The cross-sectional design does not allow to infer a causal-effect relationship between AIP and other cardiovascular risk factors. This needs a longitudinal study. We did single measurements of the laboratory parameters that could have affected accuracy of the results. The study enrolled only those patients present at the clinic during the study period and this could have caused a selection bias. We did not screen our patients for HIV and this could have affected our results.

## Conclusion

The present study showed that AIP was significantly elevated and correlated significantly with waist-hip ratio and atherocardiovascular indices among T2D patients in Uganda. AIP at no added cost can be used to stratify patients in a poor resource setting so that those at high risk can be started on primary CVD management with appropriate medications and life style programs.
